# Comparison of Chemotherapy vs Chemotherapy Plus Total Hysterectomy for Women With Uterine Cancer With Distant Organ Metastasis

**DOI:** 10.1001/jamanetworkopen.2021.18603

**Published:** 2021-07-28

**Authors:** Yuefeng Wang, Todd Tillmanns, Noam VanderWalde, Bradley Somer, Ari VanderWalde, Lee Schwartzberg, Matthew T. Ballo

**Affiliations:** 1 Department of Radiation Oncology, West Cancer Center and Research Institute, Memphis, Tennessee; 2 Department of Gynecologic Oncology, West Cancer Center and Research Institute, Memphis, Tennessee; 3 Department of Hematology/Oncology, West Cancer Center and Research Institute, Memphis, Tennessee

## Abstract

This cohort study evaluates the overall survival for patients with uterine cancer with distant organ metastasis treated with chemotherapy alone vs chemotherapy plus total abdominal hysterectomy.

## Introduction

Uterine cancer is the most common gynecologic cancer, and 9% of patients have metastatic disease at initial presentation.^1^ In addition to systemic therapy, total abdominal hysterectomy (TAH) with maximal cytoreduction has been shown to increase survival for patients with abdominal or pelvic metastases.^1,2,3^ However, to our knowledge, the role of TAH for uterine cancer with distant organ metastasis has not been established. In addition, there is growing evidence that definitive local therapies may increase survival for some types of metastatic cancers.^4,5,6^ In this cohort study, we evaluate the overall survival for patients with uterine cancer with distant organ metastasis treated with chemotherapy alone vs chemotherapy plus TAH.

## Methods

This study was approved by the West Cancer Center and Research Institute institutional review board. All patient data were deidentified in the National Cancer Database (NCDB) and, therefore, informed consent was not required, in accordance with 45 CFR §46. This study follows the Strengthening the Reporting of Observational Studies in Epidemiology (STROBE) reporting guideline.

The NCDB was used to identify patients with newly diagnosed uterine cancer with metastasis to the brain, lung, liver, bone, or distant lymph node. All patients received chemotherapy with or without TAH. Patients who received no treatments, definitive pelvic radiotherapy (dose ≥45 Gy), or those missing baseline variables were excluded (eFigure in the Supplement). Overall survival was analyzed using the Kaplan-Meier method, log-rank test, Cox proportional hazards models, landmark analysis, and propensity score–matched analyses. In all these analyses, 16 variables were used, including TAH, age, year of diagnosis, race, comorbidity score, grade, clinical T/N stage, facility type, insurance, histology, metastatic site, number of metastatic sites, hormone therapy, urban vs rural residence, education, and annual income. Race and ethnicity were analyzed in this study because they are associated with differences in cancer survival. Race and ethnicity reported in the NCDB were extracted from patients’ medical records. Subgroup survival analyses were done by age, comorbidity score, T/N stage, grade, histology, metastatic site, and number of sites. Statistical significance was calculated with 2-sided χ^2^ tests and was defined as *P* < .05. All statistical analyses were done using SAS statistical software version 9.4 (SAS Institute). This study was performed from January to June 2018.

## Results

From 2010 to 2014, we identified 3197 patients (mean [SD] age, 61.9 [11.2] years; all women [100%]) with uterine cancer with distant organ metastasis in the NCDB. Most of these patients had lung metastasis (1544 patients), followed by liver metastasis (851 patients), lymph node metastasis (497 patients), bone metastasis (249 patients), and brain metastasis (56 patients). Among these patients, 1809 received chemotherapy alone and 1388 received chemotherapy plus TAH. At a median (interquartile range [IQR]) follow-up of 13.4 (1.9-54.9) months, TAH plus chemotherapy was associated with improved survival by both univariable (hazard ratio [HR], 0.57; 95% CI, 0.53-0.62) and multivariable (HR, 0.59; 95% CI, 0.54-0.65) analysis compared with chemotherapy alone (Figure and Table). Propensity score–matched analysis demonstrated superior survival (median [IQR], 19.8 [18.3-22.3] months vs 11.0 [10.0-12.2] months; HR, 0.59; 95% CI, 0.53-0.65) for TAH plus chemotherapy. Sequential landmark analysis demonstrated significant improvement in survival for long-term survivors at greater than or equal to 0.5 year (HR, 0.69; 95% CI, 0.63-0.75), greater than or equal to 1 year (HR, 0.78; 95% CI, 0.69-0.88), and greater than or equal to 2 years (HR, 0.73; 95% CI, 0.59-0.91). On subgroup analyses, TAH plus chemotherapy was associated with significantly improved survival vs chemotherapy alone for all subgroups except patients with leiomyosarcoma (HR, 0.72; 95% CI, 0.51-1.02) or metastasis to brain (HR, 0.47; 95% CI, 0.07-3.16). Among surgical patients, 79% (1091 of 1388 patients) underwent TAH followed by chemotherapy and had significantly better survival than patients receiving chemotherapy alone (median [IQR] survival, 18.8 [17.0-20.4] months vs 10.3 [9.7-11.2] months) (Table). In the NCDB, we identified 228 patients who received definitive pelvic radiotherapy and 143 patients who underwent TAH and radiotherapy, in addition to chemotherapy (Table). Both groups of patients also had improved survival over chemotherapy alone (HR, 0.60; 95% CI, 0.51-0.71 and HR, 0.34; 95% CI, 0.26-0.44).

**Figure.**
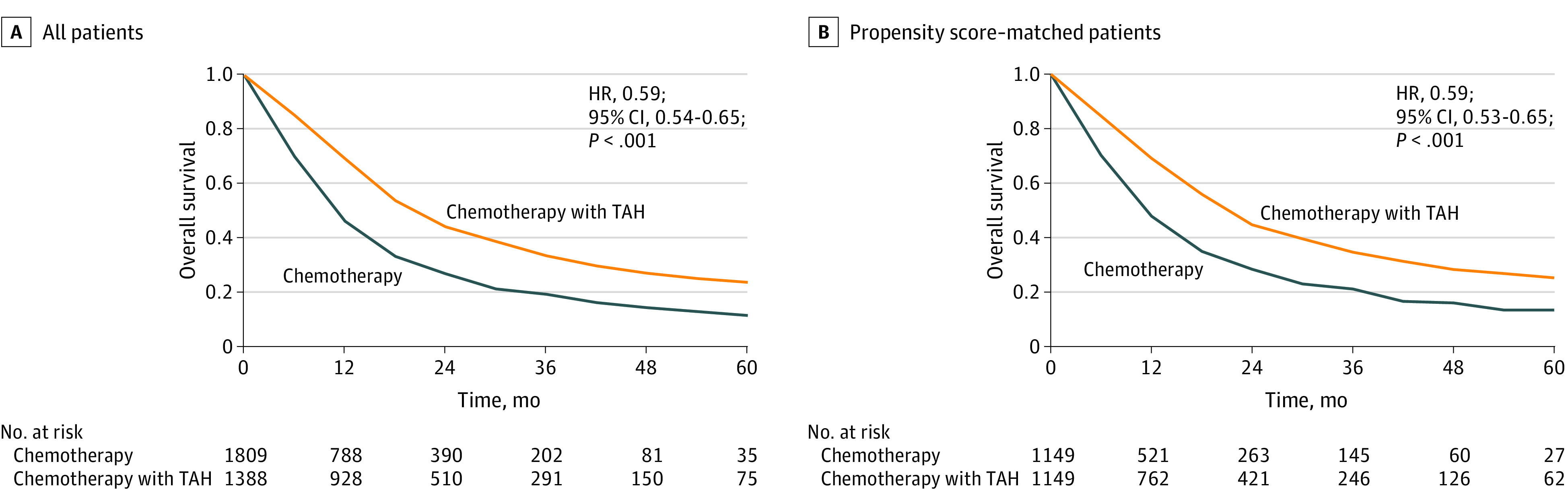
Overall Survival Among Patients With Distant Metastatic Uterine Cancer Who Received Chemotherapy Alone vs Chemotherapy Plus Total Abdominal Hysterectomy (TAH) Panel A shows survival curves for all 3197 patients. Panel B shows survival curves for 2298 propensity score–matched patients. HR indicates hazard ratio.

**Table.** Survival Analysis for All Patients and Propensity Score–Matched Patients

Treatment	Patients, No.	Survival, median (95% CI), mo	2-y OS, mean (SD), %	Univariable analysis		Multivariable analysis	
				HR (95% CI)	*P* value	HR (95% CI)^a^	*P* value
All patients							
Chemotherapy alone	1809	.3 (9.7-11.2)	26.8 (1.1)	1 [Reference]	NA	1 [Reference]	NA
TAH plus chemotherapy	1388	19.9 (18.6-21.8)	44.1 (1.3)	0.57 (0.53-0.62)	<.001	0.59 (0.54-0.65)	<.001
Chemotherapy followed by TAH^b^	297	27.6 (21.6-30.7)	52.0 (3.0)	0.47 (0.41-0.55)	<.001	0.47 (0.40-0.55)	<.001
TAH followed by chemotherapy^b^	1091	18.8 (17.0-20.4)	41.9 (1.5)	0.61 (0.55-0.66)	<.001	0.64 (0.58-0.70)	<.001
Definitive pelvic RT plus chemotherapy	228	19.9 (18.6-21.8)	42.9 (3.2)	0.56 (0.47-0.66)	<.001	0.60 (0.51-0.71)	<.001
TAH with RT plus chemotherapy	143	54.8 (34.8-not reached)	68.0 (3.8)	0.29 (0.22-0.37)	<.001	0.34 (0.26-0.44)	<.001
Propensity score–matched patients^c^							
Chemotherapy alone	1149	11.0 (10.0-12.2)	27.9 (1.3)	1 [Reference]	NA	1 [Reference]	NA
TAH plus chemotherapy	1149	19.8 (18.3-22.3)	44.4 (1.6)	0.59 (0.54-0.65)	<.001	0.59 (0.53-0.65)	<.001
Chemotherapy followed by TAH^b^	254	27.6 (21.4-32.4)	52.1 (3.3)	0.49 (0.41-0.58)	<.001	0.45 (0.38-0.54)	<.001
TAH followed by chemotherapy^b^	895	18.5 (16.7-20.1)	42.2 (1.7)	0.63 (0.57-0.70)	<.001	0.63 (0.57-0.70)	<.001

Abbreviations: HR, hazard ratio; NA, not applicable; OS, overall survival; RT, radiotherapy; TAH, total abdominal hysterectomy.

^a^ Multivariable HRs are adjusted for the same factors analyzed in the primary analysis, as described in the Methods section.

^b^ Sequence of treatments (chemotherapy and TAH) was determined by the number of days from diagnosis to initiation of treatments.

^c^ Propensity analysis was done by 1-to-1 nearest-neighbor matching and the caliper width was 0.05 times the SD of the logit of the propensity score.

## Discussion

Palliative TAH was included in the 2021 NCCN guideline for uterine cancer with distant organ metastasis. However, the role of TAH as a definitive treatment approach has not been established. To our knowledge, this analysis represents the largest reported cohort of patients with metastatic uterine cancer treated by local therapies.

To account for potential selection biases between responders and nonresponders (ie, immortal time bias), sequential landmark analysis demonstrated significant improvement in survival for long-term survivors, which suggests that the benefit of TAH in the study is not just associated with bias. In addition, by using the time of treatments initiation, we found that most (79%) surgical patients underwent TAH followed by chemotherapy and had significantly better survival than patients receiving chemotherapy alone, which helped to rule out the selection bias that TAH was only delivered to patients who had good response from neoadjuvant chemotherapy.

The median survival for patients with stage IVB uterine cancer receiving systemic therapy is less than 1 year.^3^ In this study, definitive local therapy (TAH) was associated with significantly improved survival compared with chemotherapy alone. We identified patients who received definitive pelvic radiotherapy and patients who underwent TAH and radiotherapy, in addition to chemotherapy, and both groups of patients also had improved survival over chemotherapy alone, which supports that definitive local therapies may benefit distant metastatic uterine cancer.

This study has several limitations. The information for number of metastatic lesions, specific chemotherapy agents, salvage therapies, performance status, and disease-specific survival is not available in the NCDB. Despite these limitations, the results in this analysis are intriguing.

In this cohort study, patients with newly diagnosed uterine cancer with distant organ metastasis receiving TAH plus chemotherapy lived substantially longer than patients receiving chemotherapy alone. Randomized clinical trials to evaluate the effect of TAH on distant metastatic uterine cancer appear to be warranted.

## Notes

Supplement. **eFigure. **CONSORT Diagram
