# Supplementary material for: Comparison of Chemotherapy vs Chemotherapy Plus Total Hysterectomy for Women With Uterine Cancer With Distant Organ Metastasis
Source: JAMA Netw Open. 2021 Jul 28;4(7):e2118603. doi: 10.1001/jamanetworkopen.2021.18603 (PMC8319754; doi:10.1001/jamanetworkopen.2021.18603)
Supplement: Supplement. — eFigure. CONSORT Diagram [file jamanetwopen-e2118603-s001.pdf]

Supplemental Online Content

Wang Y, Tillmanns T, VanderWalde N, et al. Comparison of chemotherapy vs chemotherapy plus total hysterectomy for women with uterine cancer with distant organ metastasis. *JAMA Netw Open*. 2021;4(7):e2118603. doi:10.1001/jamanetworkopen.2021.18603

eFigure. CONSORT Diagram

This supplemental material has been provided by the authors to give readers additional information about their work.

© 2021 Wang Y et al. *JAMA Network Open.*

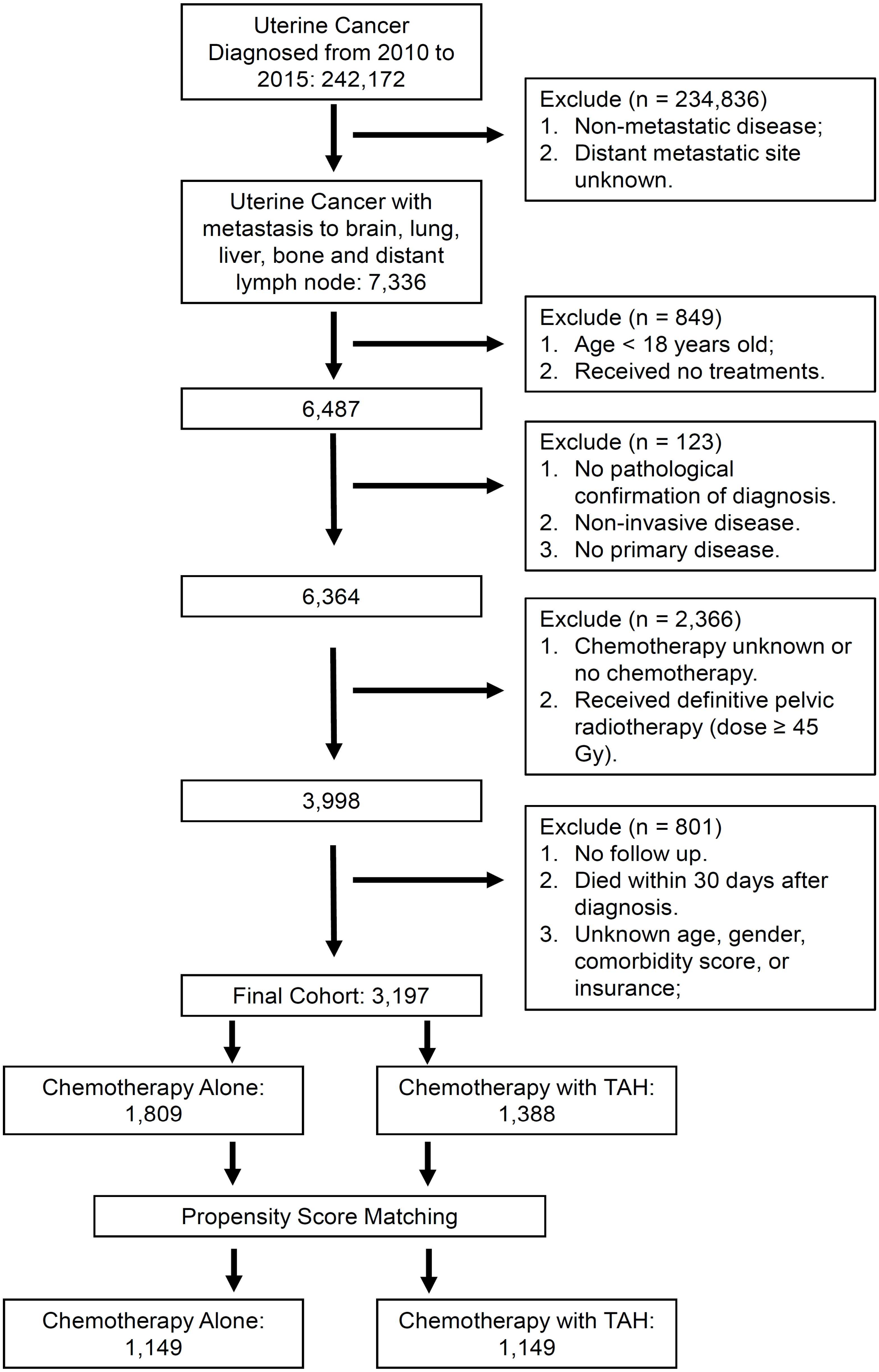

eFigure. CONSORT Diagram.

TAH, total abdominal hysterectomy.

© 2021 Wang Y et al. *JAMA Network Open*.
